# Can Theta Burst Electromagnetic Fields Disrupt Learning in Planaria? Evidence of Impaired Fear‐Conditioned Responses

**DOI:** 10.1002/bem.70017

**Published:** 2025-07-31

**Authors:** Kassra Ghassemkhani, Blake T. Dotta

**Affiliations:** ^1^ Behavioural Neuroscience & Biology Programs, School of Natural Science Laurentian University Sudbury Ontario Canada

**Keywords:** aversive stimulus, electromagnetic fields (EMFs), fear learning, memory disruption, planaria, theta burst

## Abstract

This study explored the impact of low‐intensity theta burst patterned electromagnetic fields (TBEMF) on fear‐related learning in the flatworm species Planaria, a simple model organism known for its regenerative properties and ability to demonstrate basic learning behaviors. Planaria were exposed to an aversive stimulus (light) in a T‐maze, and changes in their behavior, including time taken to select an arm and preferred arm selections, were assessed over the course of several days. The TBEMF consisted of five pulsed bursts at 100 Hz with alternating amplitudes and an intensity of 1 μT. In the group exposed to aversive light, a significant decrease in preferred arm selections was observed (*p* < 0.001), indicating that the planaria successfully learned to avoid the arm associated with the aversive stimulus. However, planaria exposed to TBEMF, either before or after the light exposure phase, did not show the same behavioral adaptation, as their arm selections remained stable, indicating that no fear learning occurred. These findings suggest that TBEMF disrupts the processes involved in fear‐related learning, likely by interfering with theta rhythm‐dependent mechanisms that are crucial for memory encoding and retrieval. Further exploration of EMF's effects on more complex organisms could reveal additional insights into its broader applications and implications for both basic neuroscience and clinical practice. Bioelectromagnetics. 00:00–00, 2025. © 2025 Bioelectromagnetics Society.

## Summary

- Planaria exposed to physiologically patterned EMFs fail to adapt to aversive stimuli, contrasting with control groups that successfully demonstrate fear learning.
- These findings suggest that physiologically patterned EMFs may interfere with theta rhythm‐dependent mechanisms, with potential implications for broader neurobiological and clinical applications.

## 1 Introduction

The electrophysiological processes of memory are highly dependent on inhibition from pacemaker neurons, which give rise to the theta rhythm (4–8 Hz) (Wulff et al. 2009; Wang 2002). This firing pattern is present in hippocampal circuits during memory encoding and retrieval (Wulff et al. 2009; Bazelot et al. 2015), dependent on the form of memory, other brain regions phase lock to hippocampal theta rhythms (Bazelot et al. 2015; Siapas et al. 2005). For instance, the amygdala phase locking to the theta band with the hippocampus either in synchrony or with gamma amplitudes (30+ Hz) is commonly reported during emotional memory processes (Bazelot et al. 2015; Costa et al. 2022). The theta wave itself acts as a carrier of information for cell populations that display fast frequency firing (Wulff et al. 2009). This can be done through the physiological process, phase‐amplitude coupling wherein fast frequency amplitudes are modulated by the phase of a slow frequency such as theta; such a pattern is present during complex cognitive processes such as memory and attentional processes (Goodman et al. 2018; Vivekananda et al. 2021; Ahn et al. 2022).

Complex low‐intensity electromagnetic fields (EMFs) can profoundly affect biological systems, influencing processes from cellular activity to broad brain region dynamics, depending on their spatiotemporal patterns (Dotta et al. 2011; Rain et al. 2024; Cook et al. 2004; Saroka and Persinger 2013; Sissons and Dotta 2024). While low‐intensity stimulation is insufficient to directly depolarize neurons, subthreshold stimulation in conjunction with voltage‐gated calcium channel modulation can alter spike duration and neurotransmitter release during subsequent depolarization (Shu et al. 2006; Lee et al. 2023; Pall 2016; Luo et al. 2014). Theta burst stimulation (TBS), a form of transcranial magnetic stimulation, is widely used to study memory and treat cognitive dysfunction (Chung et al. 2015; Sun et al. 2022; Philip et al. 2019; Noda et al. 2023; Li et al. 2022). TBS intensity is calibrated to the resting motor threshold and is typically delivered in either continuous or intermittent forms; the former inhibits cortical excitability, while the latter enhances it by modulating gamma‐aminobutyric acid (GABA) and glutamate transmission (Gamboa et al. 2011; Spurny‐Dworak et al. 2022). Notably, 40 min of TBS has been shown to disrupt fear conditioning in rats even at low intensities of 1 μT (McKay et al. 2000), and similar effects have been observed with 30 min of whole‐body exposure to EMFs patterned after hippocampal firing rhythms during long‐term potentiation (LTP) (Mach and Persinger 2009).

One hypothesis for the disruptions is through the saturation of LTP signalling pathways, optimal neuronal communication requires specific synaptic strengths (Castro et al. 1989; Beggs 2022; Moser et al. 1998). This is consistent with the concept of a neuromatrix wherein complex functions are associated with the specific spatial arrangement and electromagnetic properties of neuronal assemblies, interference with either domain can alter function, such as memory impairments and structural reorganisation arising from limbic seizure (Fournier and Persinger 2004; Holley and Lugo 2016; Choy et al. 2022). In a series of electroconvulsive therapy (ECT) experiments on patients with unipolar depression, the ability to recall memories which were reactivated before ECT were remembered less frequently than the non‐reactivated memories (Kroes et al. 2014), likely through acting on engrams that are already displaying activity, wherein the activity would make them prone to death by seizure (Naik et al. 2021). A potential advantage of low‐intensity EMFs compared to ECT for studying memory disruption is that they are noninvasive, and potentially allow for the frequency scrambling of theta rhythms in engrams without the induction of a seizure.

Rats are an extremely effective model for studies of memory given their structural overlaps with the human brain and standardised behavioural observations (Vorhees and Williams 2024; Angu Bala Ganesh 2023). The flatworm species planaria known for their regenerative properties can also be a viable cost‐effective model to study memory given they possess many of the neurotransmitters that humans possess such as acetylcholine and glutamate, also display common behavioural responses to stimuli that can be quantified, and if immobilised properly can have recordings of electric activity performed (Deochand et al. 2018; Freiberg et al. 2023; Omond et al. 2022). The planarian nervous system also hosts a bi‐lobed cephalic ganglion with a large density of multipolar neurons and a presence of dendritic spines, protrusions from dendrites which interface with axon terminals (Nakazawa 2003). Planaria also display photo‐avoidant properties and upon having UV light presented to them, will typically escape the quadrant UV light present within 60 s (Paskin et al. 2014).

A common method to study planarian memory is through the use of a Y‐shaped or T‐shaped maze, utilising either pleasant or unpleasant stimuli for conditioning (Abbott and Wong 2008). Training paradigms have shown planaria to retain memory for up to 14 days even in decapitated segments (Shomrat and Levin 2013). While theta rhythms in relation to memory and TBS effects have been extensively documented in mammals (Chung et al. 2015; Sun et al. 2022; Philip et al. 2019; Noda et al. 2023; Li et al. 2022), the pertinence to planaria has been scarcely explored.

This study aims to display planaria are capable of fear‐related learning with light exposure as the aversive stimulus, through time taken to select a maze arm and preferred maze arm selections as observable behaviours. The next aim of the experiment is to determine if theta burst patterned low intensity (1 μT) EMFs can disrupt the fear learning process through behavioural observations.

## 2 Methods

### 2.1 Aversive Stimulus Conditioning Paradigm

**Figure 1:**
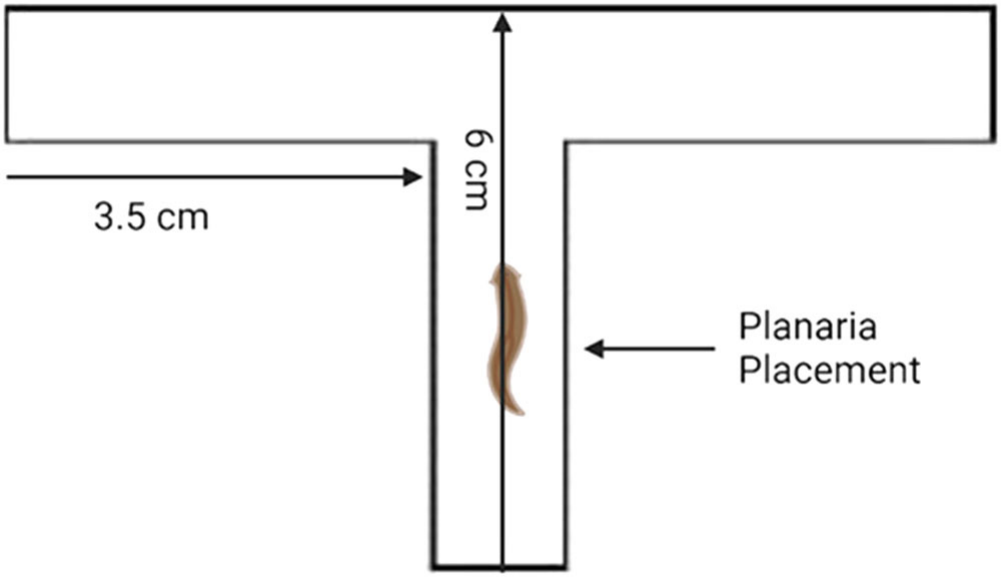
Measurements of the T‐shaped maze. Placement of the planarian is 2.5 cm in the center arm of the maze. Both the left and right arms span 3.5 cm from the center arm of the maze. Each planarian is placed facing towards the intersection of the left and right arm.

**Figure 2:**
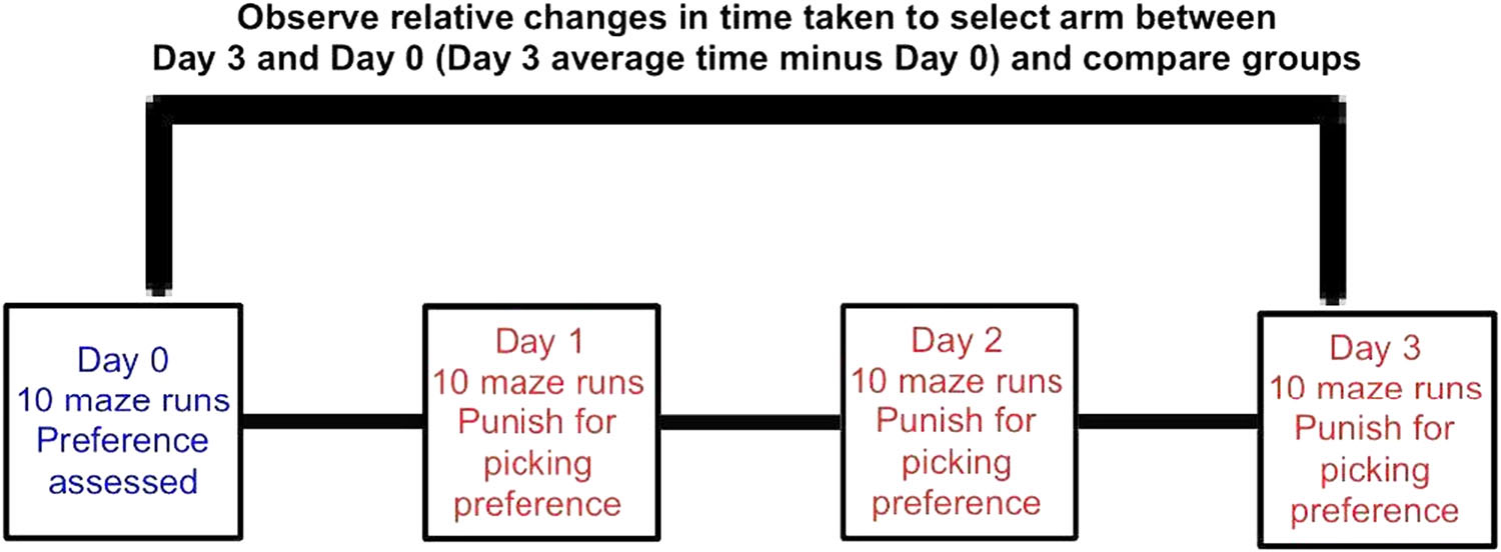
An overview of the timeline of the experiment and how relative changes in time taken are calculated. With Day 0 having the arm preference of the planarian assessed with 10 maze runs. Planaria that display an arm preference (7/10 selections) would proceed to a 3‐day conditioning paradigm with 1‐min of light exposure acting as an aversive stimulus for arm selection.

Planarian training is a 4‐day process and utilises a t‐shaped maze. Planaria are placed 2.5 cm in the centre arm of the maze facing forward and can select right or left maze arms (Figure 1). On the preconditioning day (Day 0), each planarian undergoes 10 maze runs, and directional preference is assessed. Time taken to select an arm and the arm selection are recorded in each maze run. Directional preference is determined by a minimum of 7 out of 10 maze arm selections (e.g., 7 lefts, and 3 rights). The subsequent 3 aversive stimulus conditioning days have planaria undergo the 10 maze runs on each day but receive an aversive stimulus upon selection of the preferred arm as assessed on Day 0 (Figure 2). The aversive stimulus is a 12.5 W light bulb placed 7 cm above the maze and is turned on with each preferred maze arm selection for 1 min. This 1 min of light exposure was selected as it is consistent with the photophobic avoidance responses displayed by planaria (Paskin et al. 2014). The planaria were given a 3‐min maximum per maze run to select an arm, this time frame was selected as it is standard for behavioural research in planaria, wherein around 5–30 min is when they begin to display lowered motility (Deochand et al. 2018). The planarian's entire body entering the arm qualifies as an arm selection. Upon arm selection, the planarian is carefully picked up with a transfer pipette and moved back to the centre arm. Reversal of arm preference or increased time taken to select an arm being indicative of a conditioned response.

### 2.2 EMF Application

The application of EMFs is done via a software called Complex which converts numerical values into a corresponding voltage, which are then applied as an EMF using a digital to analogue converter. The software takes a series of numbers ranging from 0 to 255 and converts them to a corresponding voltage, with any number below 128 having a negative value and anything above having a positive value. The theta burst EMF pattern is loaded onto the software. It consists of five pulsed bursts at 100 Hz with alternating amplitudes. The application of theta burst is done over a 40‐min period which is a standardised exposure time for low intensity EMF effects (Rain et al. 2024). The intensity of the field pattern is 1 μT. The field is active every 1 s with a 1 ms delay between points. The digital‐to‐analogue converter outputs to a Helmholtz coil consisting of a 39 cm × 39 cm box wrapped with insulated copper wire.

### 2.3 Conditions and Animal Housing

The planaria are grouped into four conditions: Punished, No Punishment, Theta Burst Before Punishment, and Theta Burst During Punishment. The Punished group undergoes the aversive stimulus training paradigm listed above. The No Punishment group undergoes the 4‐day process but does not receive the aversive stimulus, this group is in place to observe if arm preference retains over the 4‐day period. Planarians that receive theta burst EMF stimulation before punishment training receive the EMF immediately following the preference assessment maze runs (Day 0). The theta burst during the punishment training group receives the stimulation immediately following the Day 1 maze runs. The stock planaria are refrigerated at 4°, upon being removed from the fridge they are left at room temperature for 20 min before any usage to control for differences in locomotion immediately following refrigeration. The planaria are housed in 2 mL cuvettes in spring water at room temperature. The planaria are purchased from Boreal Science.

### 2.4 Locomotion

Given that time taken to select an arm is being assessed, a locomotion experiment was conducted to ensure that any effects on time taken were not motor features. Planaria were exposed to the same theta burst EMF pattern described above and assessed in a petri dish with 0.5 cm grid paper directly under the dish for 3 min. The open field test was conducted 24 h following EMF exposure to maintain consistency with the timeline conditioning experiment. Planarian locomotor velocity was defined as each tail crossing of a grid line.

### 2.5 Analysis

The age of the planarian stock and the size could contribute to locomotor differences between planaria. To control for size and age effects, the amount of time taken relative to Day 0 was calculated by subtracting the mean Day 0 time taken to select an arm from the mean time taken to select an arm on each of the training days. The amount of preferred arm selections is also being compared between the third days of each condition. Within the punish group, to determine if the planaria are learning, each day is compared using a paired samples *t*‐test.

## 3 Results

### 3.1 Alterations in Preferred Arm Selections Across Testing Days

**Figure 3:**
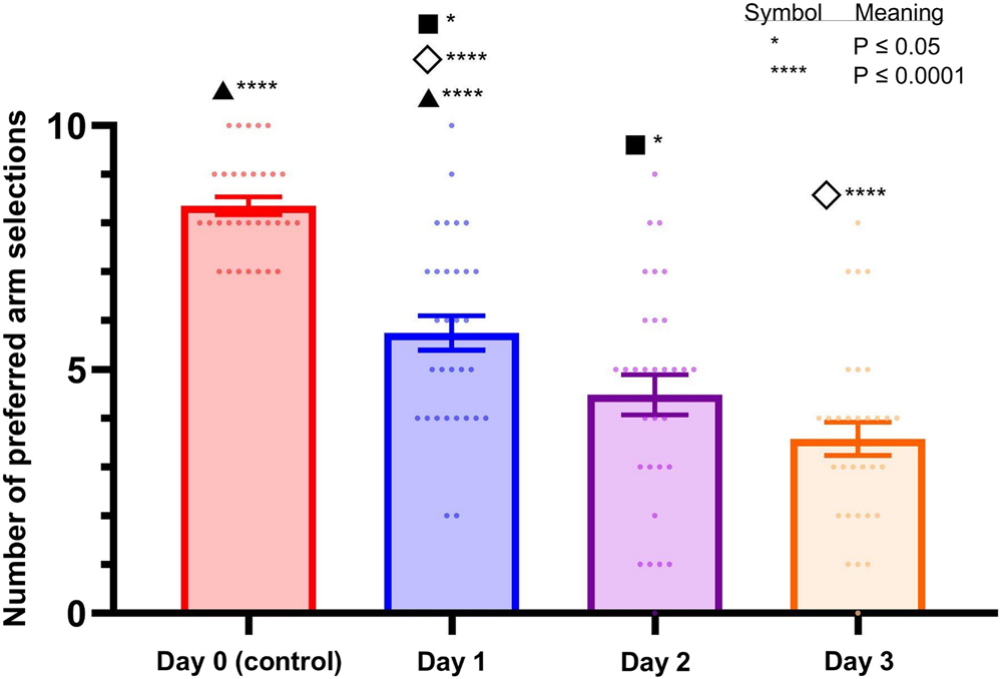
The number of preferred arm selections over 4 days of testing. Day 0 represents the control condition, while Days 1 through 3 represent days post Punishment. Statistical significance is indicated by symbols above the bars. Data are presented as mean ± SEM, with individual data points shown as dots.

The number of preferred arm selections across the 4 testing days is shown in Figure 3. A significant decrease in arm selections was observed throughout the testing period. When comparing Day 1 to Day 0 (control), Day 1 was associated with a significant decrease in preferred arm selections [*t*(30) = 13.11, *p* < 0.0001]. Similarly, Day 2 demonstrated a significant reduction in arm selections compared to Day 1 [*t*(30) = 2.623, *p* = 0.0136], and Day 3 showed a further decrease in preferred arm selections compared to Day 1 [*t*(30) = 4.883, *p* < 0.0001]. These results indicate that the experimental manipulation produced a progressive reduction in preference for the specific arm across days. The consistent reduction suggests that the aversive stimulus (light) was effective, and the animals learned to avoid the arm with the light.

### 3.2 Latency in Arm Selection

**Figure 4:**
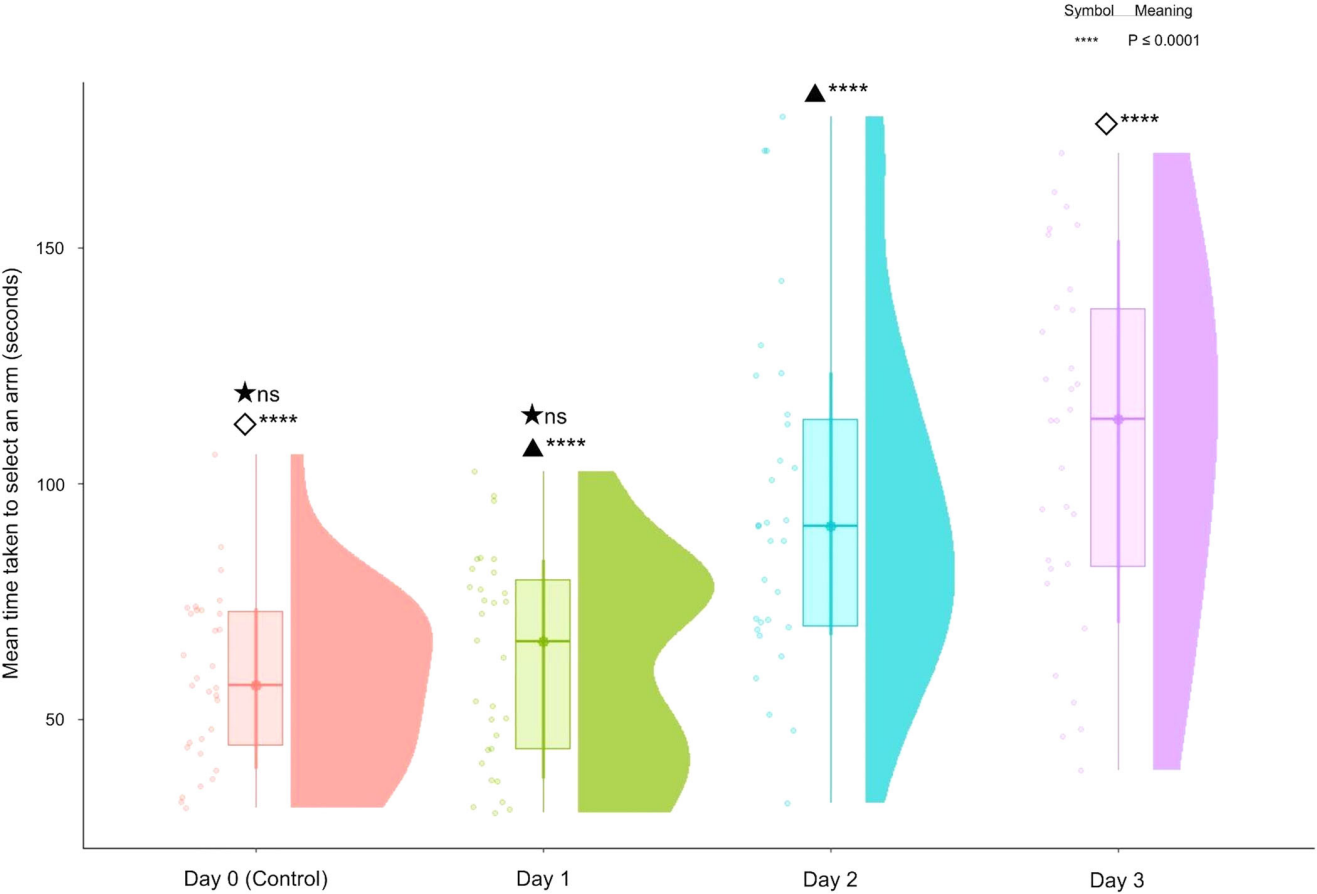
The mean time taken to select an arm across 4 days of testing. Day 0 represents the control condition, while Days 1 through 3 represent experimental conditions. Statistical significance is indicated by symbols above the box plots. Significant differences are noted with ****(*p* ≤ 0.0001), while nonsignificant differences are indicated by ‘ns’.

The mean time taken to select an arm is presented in Figure 4. There was no significant difference between Day 1 and Day 0 [(30) = 0.8186, *p* = 0.4195]. However, similar to preferred arm selection, a significant increase in time taken to enter an arm was observed from Day 2 to Day 1 [*t*(30) = 4.521, *p* < 0.0001] and from Day 3 to Day 1 [*t*(30) = 7.482, *p* < 0.0001]. This indicates a progressive delay in arm selection behavior over the duration of the testing days.

### 3.3 Arm Selections Across Different Experimental Conditions

**Figure 5:**
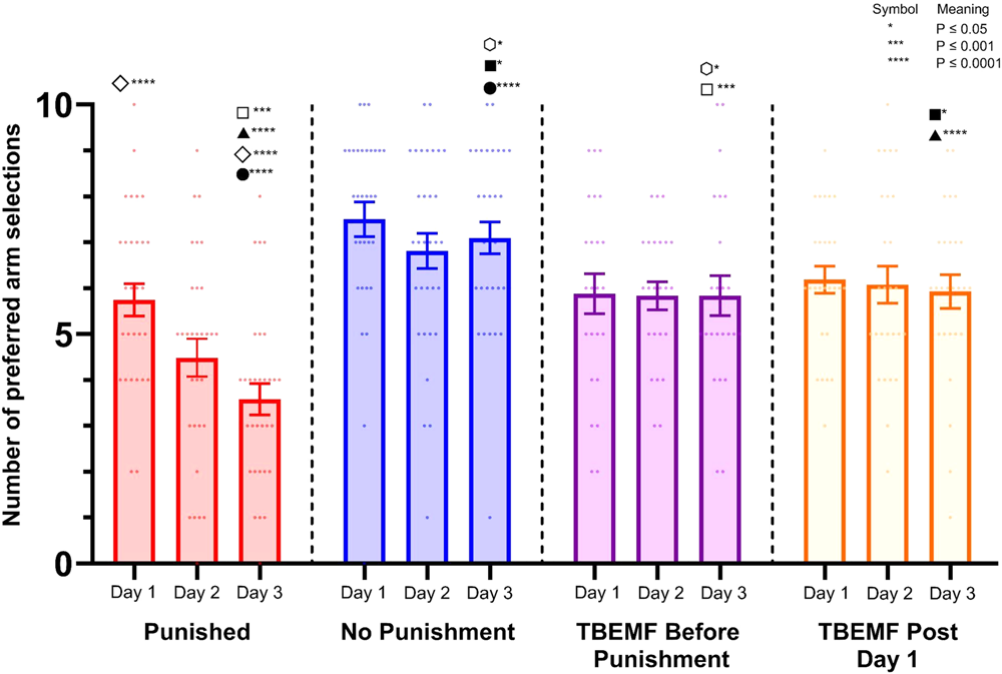
The mean preferred arm selections across three days of testing under different conditions: Punished, No Punishment, TB EMF Before Punishment, and TB EMF Post Day 1. Statistical significance is indicated by symbols above the bars. Significant differences are noted with *, ***, and **** (*p* ≤ 0.05, *p* ≤ 0.001, and *p* ≤ 0.0001, respectively). Data are presented as mean ± SEM, with individual data points shown as dots.

The mean number of preferred arm selections across the 3 days under different experimental conditions are shown in Figure 5. In the Punished group, there was a significant decrease in arm selections across days (*p* ≤ 0.0001), while the No Punishment group exhibited no significant changes. In both the Theta Burst EMF (TBEMF) Before Punishment and TBEMF Post Day 1 conditions, no significant changes were observed across days, suggesting that the TBEMF manipulation prevented learning of the aversive stimulus. Animals in these conditions selected the aversive arm as frequently as in the control conditions, unlike those in the Punished group who did not receive TBEMF.

### 3.4 Time Taken to Select an Arm Across Different Experimental Conditions

**Figure 6:**
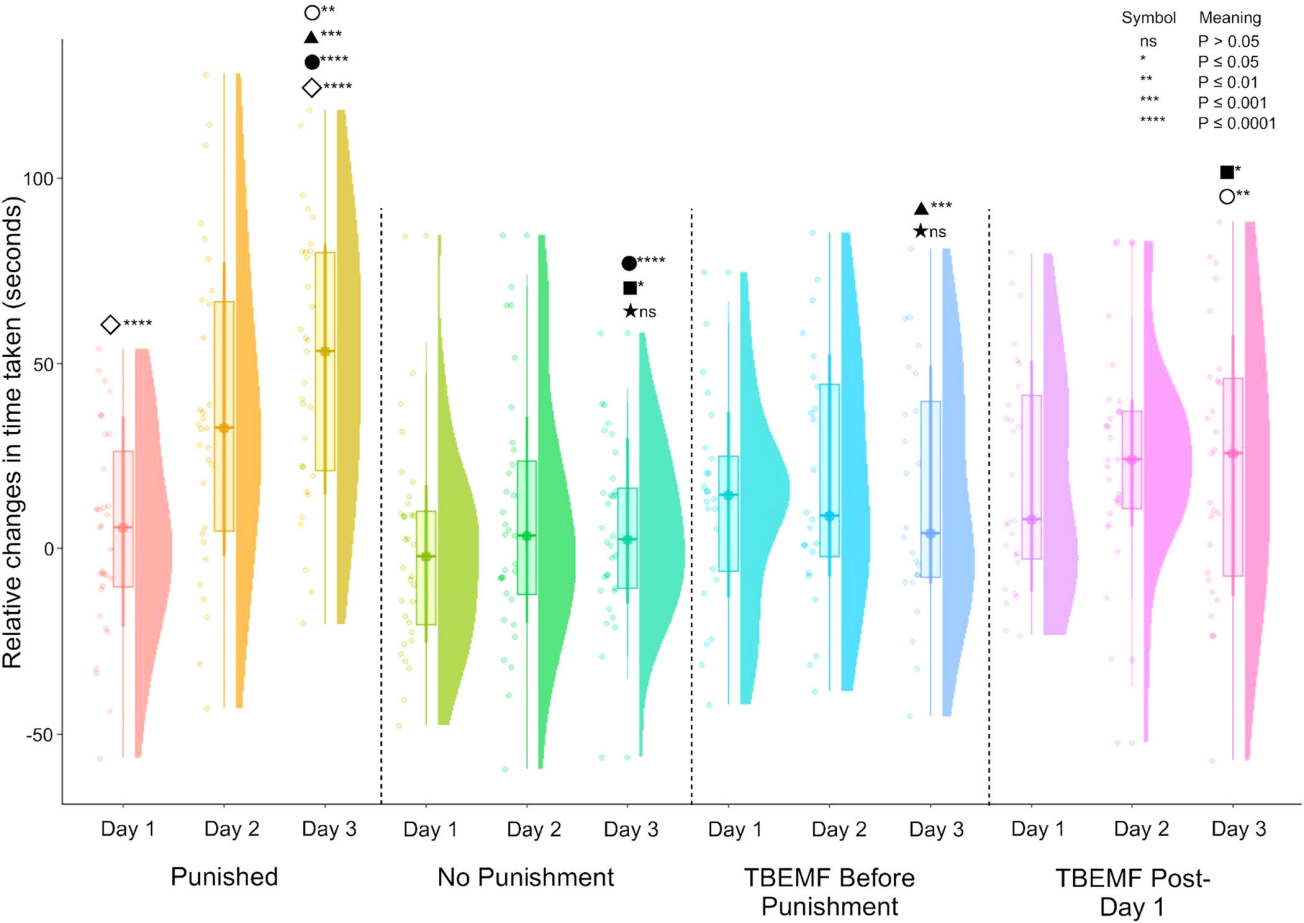
Relative changes in time taken to select an arm across three days of testing under different conditions. Statistical significance is indicated by symbols above the box plots. Significant differences are noted with ns, *, **, ***, and **** (*p* > 0.05, *p* ≤ 0.05, *p* ≤ 0.01, *p* ≤ 0.001, and *p* ≤ 0.0001, respectively). Data are presented as box plots with overlaid jittered individual data points and a density distribution.

**Figure 7:**
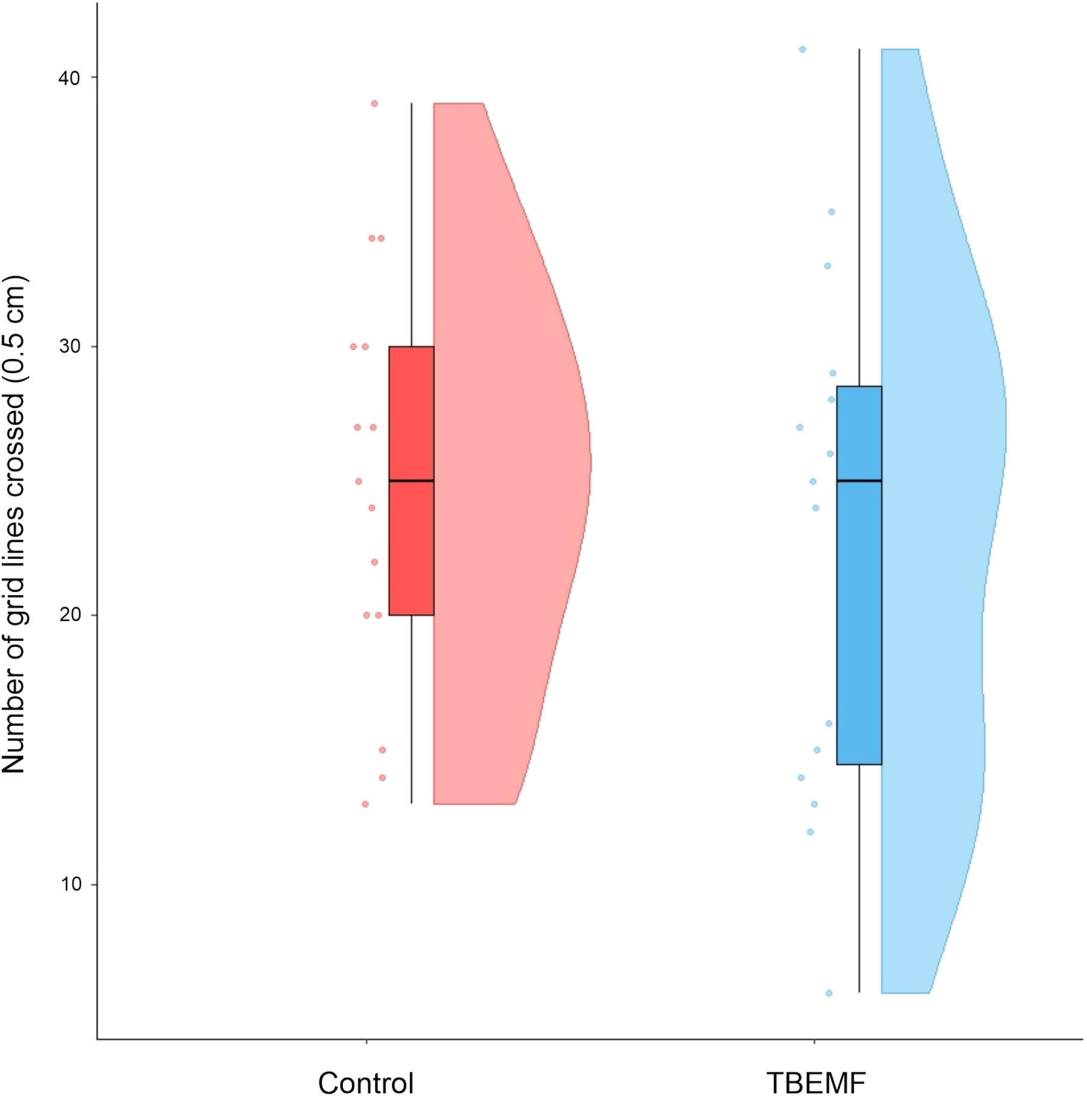
Comparison of the number of grid lines crossed between the control and theta burst conditions. The box plots display the median, interquartile range (IQR), and potential outliers, with individual data points jittered to reduce overlap. The density plots on the right side of each box plot show the distribution of data points.

Relative changes in time taken to select an arm across days under different experimental conditions are shown in Figure 6. In the *Punished group*, a significant increase in the time taken to select an arm was observed from Day 1 to Day 2 (*p* < 0.0001) and from Day 1 to Day 3 (*p* < 0.0001). The *No Punishment group* showed no significant changes in time taken across days. However, in both the *TBEMF Before Punishment* and *TBEMF Post Day 1* groups, a significant increase in time taken was observed across days (*p* < 0.05). This shows that the TBEMF application groups were similar to the *No Punishment* conditions, indicating that no learning of the aversive stimulus occurred. This finding is consistent with the arm selection data, where no significant changes in preference were observed in the TBEMF groups. To ensure that these effects were not a simple increase in mobility effects, Figure 7 compares the number of grid lines crossed between the *Control* and *TBEMF Conditions*. While there was a nonsignificant reduction (*p* > 0.05) in the number of grid lines crossed in the *TBEMF* condition, indicating that TBEMF stimulation did not significantly affect overall locomotor activity. Additionally, means and standard deviations of mean time taken and mean preferred arm selections across the different experimental groups are shown in Table 1.

**Table 1:** Summary of mean time taken (in seconds) and mean preferred arm selections across different groups over four phases: Pre‐Training, Day 1, Day 2, and Day 3.

	*n*	Pre‐Training	Day 1	Day 2	Day 3
**Mean time taken**					
Groups					
Fear conditioned	31	58.87 ± 18.04	62.95 ± 21.97	94.96 ± 36.13	108.40 ± 37.49
No fear conditioning	32	57.49 ± 19.21	56.59 ± 28.87	65.09 ± 32.53	62.31 ± 22.09
TBS before fear conditioning	24	60.17 ± 20.06	72.90 ± 26.80	79.10 ± 29.14	74.75 ± 26.66
TBS post Day 1	27	57.62 ± 18.82	77.46 ± 23.48	80.94 ± 24.49	79.91 ± 28.65
**Mean preferred arm selections**					
Groups					
Fear conditioned	31	8.355 ± 1.018	5.742 ± 1.949	4.484 ± 2.293	3.581 ± 1.911
No fear conditioning	32	8.313 ± 1.176	7.500 ± 2.140	6.813 ± 2.177	7.094 ± 1.957
TBS before fear conditioning	24	8.417 ± 1.100	5.875 ± 2.133	5.833 ± 1.494	5.833 ± 2.140
TBS post Day 1	27	8.000 ± 0.9607	6.185 ± 1.520	6.074 ± 2.093	5.926 ± 1.899

## 4 Discussion

The results of this study provide important insights into the effects of theta burst patterned low‐intensity electromagnetic fields (TBEMF) on planarian learning and behavior. The primary aim was to establish whether planaria, when exposed to an aversive stimulus (light), would exhibit fear‐related learning as measured through their preferred maze arm selections and the time taken to select an arm. Additionally, we investigated whether TBEMF exposure would disrupt this learned behavior.

Our data demonstrate that planaria are capable of fear‐related learning, as indicated by the significant reduction in preferred arm selections across days in the Punished condition. The presentation of light as an aversive stimulus led to a consistent decrease in the selection of the illuminated arm. These findings support the notion that planaria possess the ability to associate negative stimuli with specific environmental cues and adjust their behavior accordingly, consistent with previous research on planarian learning capabilities using aversive stimuli. The progressive decrease in arm selections in the punished group suggests that planaria learned to avoid the aversive stimulus (light) over time. This type of behavior mirrors memory and learning processes observed in more complex organisms and aligns with earlier studies on memory retention in planaria (Abbott and Wong 2008; Shomrat and Levin 2013). Additionally, the observation that planaria exhibited no significant changes in arm selections in the No Punishment group further reinforces the idea that the changes observed in the punished group were due to the aversive nature of the stimulus rather than spontaneous behavior.

In contrast to the punished group, both TBEMF groups did not show the same reduction in arm selections over time, indicating that TBEMF exposure disrupted the fear learning process. The animals exposed to TBEMF continued to select the aversive arm at rates similar to the control and no punishment conditions, suggesting that the electromagnetic field exposure interfered with the encoding or retrieval of the aversive stimulus‐memory association. This observation is consistent with previous studies indicating that low‐intensity EMFs can disrupt cognitive and learning processes. The lack of behavioral adjustment in the TBEMF groups suggests that the patterned electromagnetic fields scrambled or interfered with the neural processes required for memory formation and retrieval in planaria. One possible explanation for this disruption is the interference of TBEMF with the cellular mechanisms underlying synaptic plasticity, specifically long‐term potentiation (LTP), which has been shown to be modulated by theta rhythms (Spurny‐Dworak et al. 2022; Mach and Persinger 2009; Hölscher et al. 1997a; Etter et al. 2023).

The results of this study extend the body of research on the effects of electromagnetic fields on biological systems, particularly on learning and memory (Sun et al. 2022; Philip et al. 2019; McKay et al. 2000; Mach and Persinger 2009). Previous research has demonstrated that low‐intensity electromagnetic fields can affect a variety of neural and cognitive processes, including memory and sensory perception (Dotta et al. 2011; Cook et al. 2004; Saroka and Persinger 2013). Our data add to this literature by providing evidence that TBEMF specifically disrupts fear learning in planaria. The potential mechanism underlying this disruption may involve the modulation of phase‐amplitude coupling and other neural processes associated with theta rhythms (Spurny‐Dworak et al. 2022; Etter et al. 2023). Theta burst stimulation has been shown to influence LTP in mammalian systems (Capocchi et al. 1992; Rodrigues et al. 2021), and this study suggests that similar effects may be at play in simpler organisms such as planaria. The planarian nervous system, while less complex, shares certain neurotransmitter systems and neural structures with higher organisms (Omond et al. 2022; Nakazawa 2003), making them a viable model for exploring the effects of TBEMF on learning and memory.

Theta rhythms play a critical role in facilitating long‐term potentiation (LTP), as demonstrated by stimulation in CA1 inducing 90% of an excitatory postsynaptic potential (EPSP) amplitude during the positive phase of theta, effectively generating LTP (Hölscher et al. 1997b). LTP involves synaptic strengthening through the insertion of α‐amino‐3‐hydroxy‐5‐methyl‐4‐isoxazolepropionic acid (AMPA) receptors, which depolarize neurons and enable the removal of the magnesium block from N‐methyl‐d‐aspartate (NMDA) receptor pores (Malenka and Bear 2004; Sumi and Harada 2020). This process allows calcium influx, triggering downstream targets like CaMKII to support trafficking of NMDA and AMPA receptors, tubulin phosphorylation, and actin remodeling (Sumi and Harada 2020; Craddock et al. 2012; Bonilla‐Quintana and Wörgötter 2021). Furthermore, optogenetic activation of the medial septum, a cholinergic pacemaker for hippocampal theta rhythms, has been shown to disrupt hippocampal theta oscillations and impair memory retrieval (Etter et al. 2023).

Aberrant network connectivity is a hallmark of posttraumatic stress disorder (PTSD), often manifesting as heightened physiological responses to aversive memory cues following trauma (Bryant 2019). For example, PTSD is associated with increased connectivity between fear‐related structures, such as the periaqueductal gray, and the default mode network, which governs self‐referential thought (Terpou et al. 2020). Connectivity changes are also observed in the salience network, including heightened interactions between the amygdala and hippocampus (Neumeister et al. 2017; Osuch et al. 2008). Intermittent TBS has demonstrated therapeutic potential by increasing connectivity within default mode network structures and improving clinical scale scores in PTSD patients (Philip et al. 2019).

While the current findings are intriguing, several limitations should be acknowledged. First, the planarian nervous system is relatively simple compared to mammals, so the mechanisms underlying their response to TBEMF may not fully translate to more complex organisms. However, planaria share key features such as neurotransmitter systems and basic neural structures, making them a valuable model for studying fundamental learning and memory behaviors. By using planaria, this study provides a cost‐effective and ethically sound approach to investigate the behavioral effects of low‐intensity EMFs. These findings help to elucidate how low‐intensity EMFs can disrupt fear‐related learning, offering a foundation for future research in more complex organisms, including vertebrates and humans. Further studies utilizing electrophysiological recordings in planaria could help clarify how TBEMF affects neural circuits during learning and memory processes. Additionally, the study focused on a single intensity of TBEMF (1 μT). Future research should explore a range of field intensities and patterns to determine whether these variables influence the degree of disruption observed. Moreover, expanding the experimental design to include long‐term memory tests could provide insights into whether the effects of TBEMF on memory are transient or permanent.

## 5 Conclusion

In conclusion, this study demonstrates that planaria are capable of learning to avoid an aversive stimulus and that TBEMF exposure disrupts this learning process. The significant reduction in arm selections in the punished group indicates that planaria can adapt their behavior in response to negative stimuli, while the TBEMF groups showed no such learning. These findings suggest that TBEMF interferes with the neural mechanisms underlying fear learning in planaria, likely by affecting processes dependent on theta rhythms, which are crucial for memory encoding and retrieval across species. Given the growing interest in the effects of low‐intensity EMFs on brain function and behavior, this study contributes to a broader understanding of how electromagnetic fields can modulate cognitive processes. Future research should focus on further elucidating the specific mechanisms by which TBEMF disrupts memory and learning, both in planaria and more complex organisms. This knowledge could inform potential therapeutic applications where controlled memory disruption is desirable, while also highlighting the need to understand the long‐term effects of EMF exposure on cognitive function.

## Conflicts of Interest

The authors declare no conflicts of interest.
